# Chimney Graft Technique Combined With Embolization for Treating Ruptured Aortic Arch Lesions

**DOI:** 10.3389/fcvm.2021.711283

**Published:** 2021-10-04

**Authors:** Xianhao Bao, Yuxi Zhao, Tao Li, Mingwei Wu, Zhaoxiang Zeng, Minxin Gao, Ding Xu, Jiaxuan Feng, Rui Feng

**Affiliations:** ^1^Department of Vascular Surgery, Shanghai General Hospital, Shanghai Jiao Tong University School of Medicine, Shanghai, China; ^2^Department of Cardiovascular Surgery, Jinling Hospital, Medical School of Nanjing University, Nanjing, China

**Keywords:** chimney graft, embolization, ruptured aortic arch lesions, endoleak, endovascular treatment

## Abstract

**Background:** This study aimed to share the experience in applying the chimney graft technique combined with embolization for treating aortic arch rupture under emergency conditions and evaluating early-term results in these patients.

**Methods:** This study retrospectively included patients with ruptured aortic arch lesions who received the chimney graft technique combined with embolization between March 2016 and March 2021. The primary endpoint was a technical success, deemed as successful stent graft deployment to the planned location, patency of the target branch vessel, and absence of significant type I endoleak. The secondary endpoint was clinical success defined with the size of false lumen in follow-up remaining unchanged or decreasing over time, 30-day mortality, complication, and primary patency of chimney graft.

**Results:** This study included 12 patients (age, 61 ± 12 years; male, 83%). Five patients (42%) received single chimney, one patient (8%) received double chimney, and six patients (50%) received triple chimney. Intraoperative type I endoleak occurred in six patients (50%) who underwent endovascular embolization in the primary operation. Post-operative type I endoleak, evaluated by computed tomography angiography examination following the primary operation, occurred in seven patients (58%), including one patient who received endovascular embolization two times. All patients with post-operative type I endoleak were successfully re-treated using coil and Onyx glue within 1 week, and the median length of stay was 22 ± 11 days (range: 7–44 days). Overall technical success was 100%. Eleven patients had completed their follow-up (median, 12 months, range: 1–34 months), and one patient was out of contact. The 30-day mortality was 9% (1/11, post-operative death of a patient with cerebral hemorrhage). No major complications and no chimney compression, migration, occlusion, or stenosis were recorded during follow-up. Seven patients (58%) have ≥6 months of clinical follow-up time with appropriate imaging. In four (57%) of these patients, diameter stabilization was detected, whereas three (43%) experienced significant reduction (≥5 mm).

**Conclusion:** The patients in this study had satisfactory early-term outcomes. The chimney graft technique combined with coil and Onyx glue embolization may be a safe and effective treatment for ruptured aortic arch lesions under emergency conditions.

## Introduction

Aortic arch rupture is an extremely rare but potentially fatal condition. Open surgery remains the standard strategy for this emergency. However, some patients with advanced age and/or multiple organ dysfunction cannot tolerate the traditional open repair (1–3). Due to limited proximal anchoring area for ruptured aortic arch lesions, hybrid technique is safe and reliable in dealing with arch pathologies. However, it still had certain operative trauma because of original anatomical structure damage (4, 5). Thoracic endovascular aortic repair (TEVAR) has been widely used to apply innovative techniques or remodeled stent grafts, including fenestrated grafts, scallop stent grafts, branch grafts, and chimney grafts (CGs) (6–8). Although this approach is minimally invasive, it usually requires customization for each patient due to the complexity and variability of the superior branch of the aortic arch.

In the CG technique, standardized and ready-made stent grafts are used, allowing for emergency treatment of aortic rupture lesions with insufficient sealing zones (9). CGs extend the sealing zone of thoracic aortic stent graft to a certain extent and ensure blood flow to vital branch arteries (10–12). Therefore, CG technique can be an urgent choice for patients with serious complications unsuitable for open surgery (13, 14). However, due to a lack of customized stent grafts and technical guidelines, the result of CG technology is not always satisfactory. According to reports, the occurrence of type I endoleak (EL-I) following chimney-TEVAR (cTEVAR) can be as high as 18%−50% (15–18). For ease of description, the sac outside the aortic stent graft is temporarily defined as a false lumen (FL). The gutter between aortic stent graft and CGs might allow blood to enter FL, leaving the risk of rupture or bleeding of aortic arch unsettled. The deployment of coil and Onyx glue to induce thrombosis in FL and gutters based on the CG technique is a key strategy to solve this problem, which is feasible and effective based on our experience in treating aortic arch rupture patients (19, 20).

## Materials and Methods

### Patients

Between March 2016 and March 2021, our center treated 34 patients with ruptured aortic arch lesions. The inclusion criteria for this study were as follows: (1) ruptured aortic arch lesions were diagnosed using computed tomography angiography (CTA) or digital subtraction angiography (DSA); (2) first onset, or was treated with open surgery or TEVAR, but the treatment failed; (3) CG and embolization techniques were used in the hospitalization process. Exclusion criteria were as follows: (1) intramural hematoma and aortic penetrating ulcer; (2) reconstruction of superior branches of aortic arch through cervical or intra-thoracic vascular bypass combined with TEVAR; (3) during operation, other TEVAR techniques such as *in situ* fenestration, slot, and branch were used; (4) both techniques described in this study were applied in operation but without thrombosis of FL or gutters. A total of 12 patients who underwent therapeutic strategy was included in the present analysis gradually. The study protocol was approved by the local Institutional Review Board and Ethics Committee. All patients enrolled in the study provided informed consent for the procedure.

### Selection of Endovascular Prosthesis

The size and length of stent grafts used in each case were determined by information acquired from CTA and intraoperative aortography with calibrated catheters. The selection of CG type, aortic stent graft type, and embolic material was based on institutional practice and surgeon's preference, as were other technical aspects of TEVAR procedures performed. There were no device exclusions.

### The First Operation Procedure

All patients received general endotracheal anesthesia. The unilateral femoral artery was exposed through a groin oblique incision, and a 6-French catheter sheath was cannulated, from which a 5-French pigtail catheter was put into ascending aortic artery *via* a 0.035-inch guidewire before systemic heparinization. The aortic stent graft was introduced upward into the thoracic aortic arch *via* femoral artery access. The other side of femoral artery was exposed by the described method, and a catheter was preset in FL.

By taking triple chimney (TC) as an example, bilateral brachial arteries and left common carotid artery (LCCA) were exposed through an upper arm longitudinal incision and cannulated with an 8-French catheter sheath in patients who received TC. Three CGs were preset by introducing a guidewire into the ascending aorta, from the left subclavian artery (LSA), LCCA, and innominate artery (IA). Aortic arch angiography was performed to confirm the crevasse position, the branches of aortic arch, and deployment position.

**Figure 1:**
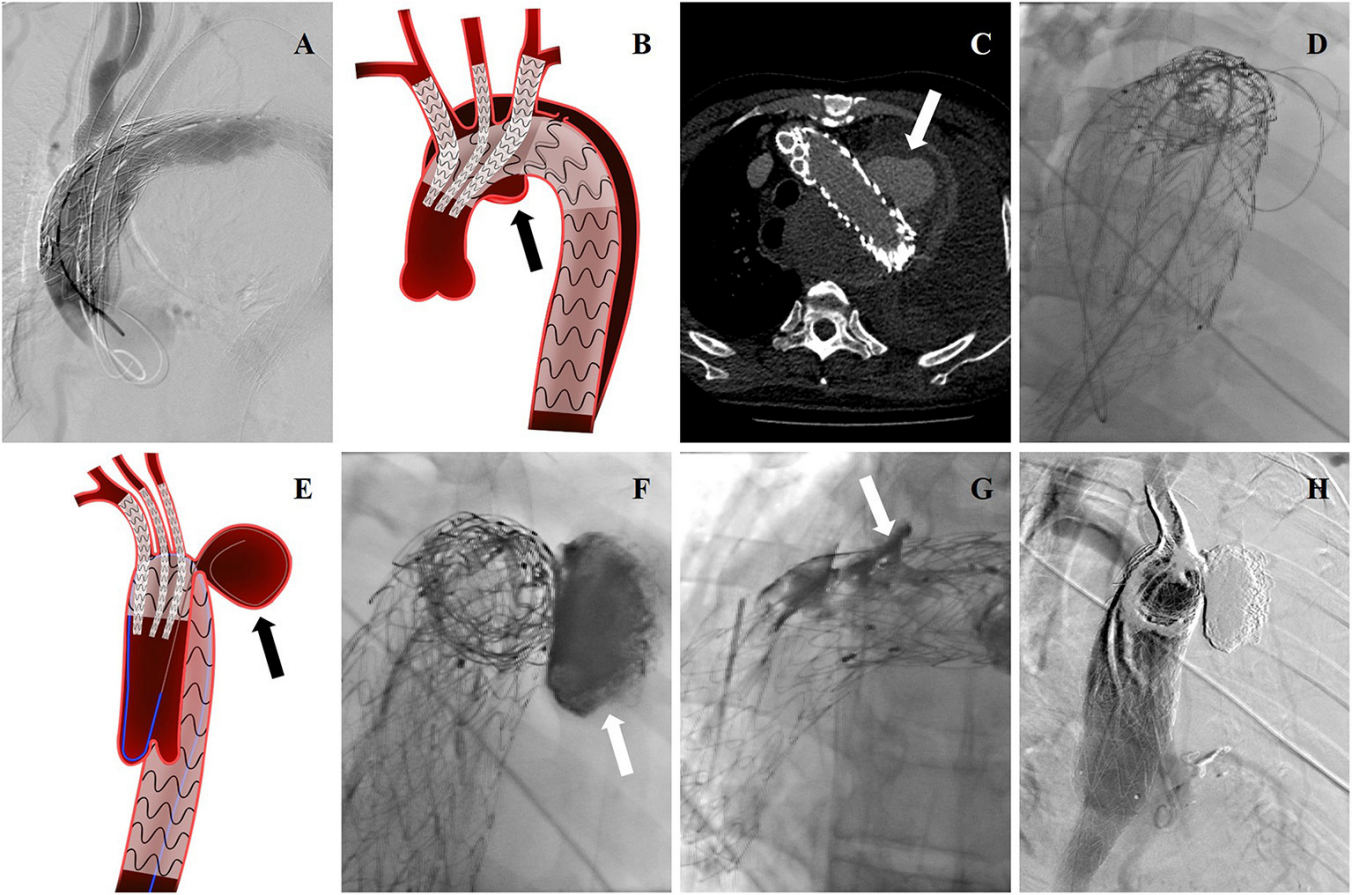
**(A)** Intraoperative DSA showing this patient was treated with TEVAR before, and a new pseudoaneurysm occurred. cTEVAR was performed in the first operation. **(B)** Endovascular treatment was shown by cartoon graph. **(C)** Post-operative EL-I was revealed by CTA reexamination, and pseudoaneurysm (arrow) almost occupied the entire left thoracic cavity. **(D)** The guidewire and catheter entered into FL through gutters by choosing femoral artery as the access artery in the second operation. **(E)** The cartoon graph demonstrated the method of selecting guidewire into FL (arrow). **(F)** Onyx glues were injected into FL through a microcatheter (arrow). **(G)** Onyx glues were injected into gutters (arrow). **(H)** Endoleak disappeared in the last aortography.

Under controlled hypotension (systolic blood pressure <90 mmHg) and fluoroscopy, the aortic stent graft was first deployed, followed by CGs. Thus, the ischemic time of superior branch of aortic arch was minimized. All proximal CGs beyond the tectorial membrane part of aortic stent graft range ~5–10 mm, whereas the distal part of CGs was deployed in respective branch arteries (Figures 1A,B). If intraoperative EL-I was found by aortography, embolic materials were immediately filled in FL or gutters between aortic stent graft and CGs through a pre-road catheter. Complete aortography confirmed the patency of arch branches and the elimination of FL without evidence of endoleak.

**Figure 2:**
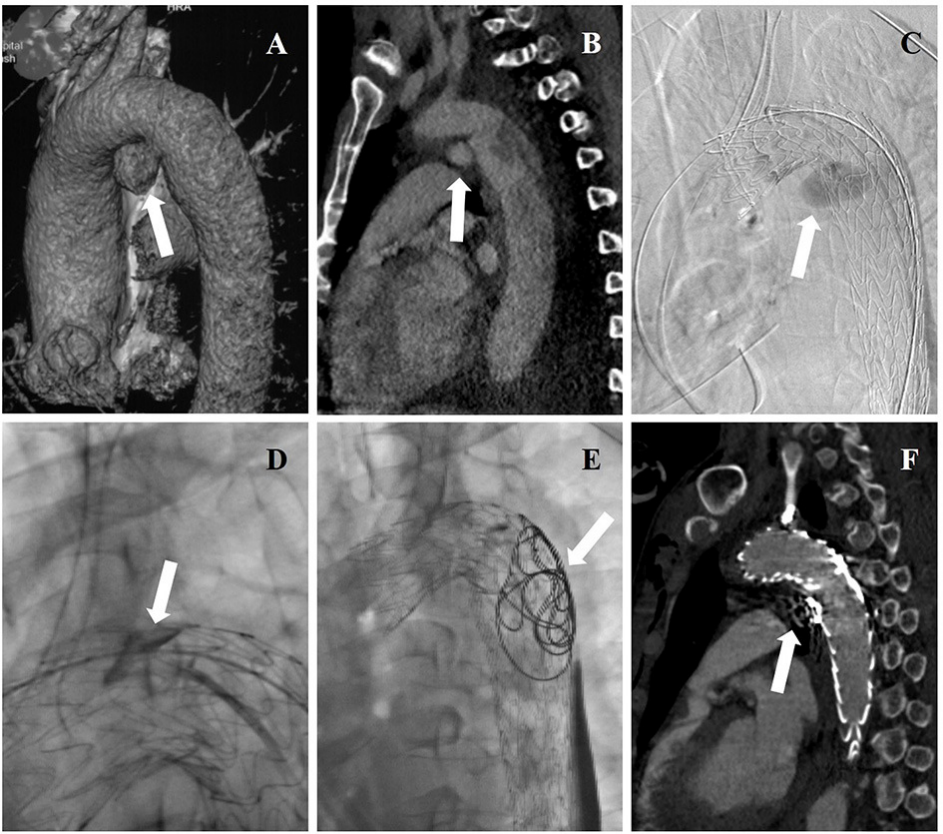
**(A)** Three-dimensional reconstructions of pre-operative CTA showing concomitant aortic arch pseudoaneurysm (arrow) of traumatic aortic dissection. **(B)** Pre-operative CTA showing the entry tear (arrow) of aortic dissection. **(C)** Intraoperative DSA showing deployment of aortic stent graft and CGs, and angiography through a pre-road catheter to FL revealed intraoperative EL-I (arrow). **(D)** Onyx glues were injected into gutters (arrow) through a microcatheter. **(E)** Coils and Onyx glues were deployed into FL (arrow) through a pre-road catheter. **(F)** The aortic dissection and pseudoaneurysm were entirely excluded while endoleak disappeared (arrow) in post-operative CTA.

### The Second Operation Procedure

Patients who complained of chest pain after the initial operation were reexamined by CTA, which revealed post-operative EL-I (Figure 1C). All patients underwent reintervention to embolize lesions and gutters. Local anesthesia was performed. The right femoral artery or the left or right brachial artery was chosen as the access artery, according to anatomic characteristics of the entry location. We took a right femoral artery as an example. After the percutaneous puncture, a 5-French pigtail catheter cannulated with a 6-French catheter sheath was placed into the ascending aortic artery *via* a 0.035 guidewire. The guidewire and catheter were promoted along the curve formed between the ascending aorta and the aortic valve and inserted into the lesions of the aortic arch or FL through selecting the gutters after the reversal (Figures 1D,E). Detachable coils were deployed into FL, where thrombosis was induced, filling the whole space. A microcatheter (Echelon-10; ev3 Neurovascular, Irvine, Calif) was introduced, through which Onyx glues (ev3 Neurovascular) were injected into FL and gutters (Figures 1F,G). The volume of injected Onyx glue was estimated according to anatomic parameters. Glue injection was stopped once gutters were fully filled, which could be observed through angiography. After injection, a final aortography was performed to confirm the patency of arch branches and elimination of EL-I (Figure 1H).

### Data Collection and Analysis

Patient demographics, characteristics of pathologies, type of stent grafts, type of embolic materials, and individual procedural details were all collected and reviewed. Three-dimensional reconstructions of pre-operative and post-operative CTA were processed by TERARECON workstation (Aquarius, CA). All measurements of aorta and branch arteries were conducted using central line model.

### Endpoints and Follow-Up

The primary endpoint was a technical success, which was deemed as successful deployment of stent graft to the planned location, patency of the target branch vessel, and absence of significant EL-I. The secondary endpoint was a clinical success with the size of false lumen in follow-up remaining unchanged or decreased, 30-day mortality, complication, and primary patency of the CG.

Physical examination, CTA scans, and 3D reconstruction were performed at 3 months, 6 months, and then annually after a second operation. The morphology, position, and patency of aortic grafts and CGs were evaluated. Thrombosis of FL or gutters was analyzed. Additional CTA examinations were obtained in patients manifesting signs or symptoms of adverse reactions. All evaluations were made by vascular surgeons and were verified independently by the first author and the coauthor.

### Statistical Analysis

All analyses were conducted using IBM SPSS 19.0 software (IBM Corp., Armonk, NY). Continuous variables in Gaussian distribution were described as mean ± standard deviations (SD). Skewed variables were summarized as median and range. The specific data were provided as the count and percentage. The diameters were compared using two-sided paired sample Student's *t*-tests. A *p*-value <0.05 was considered statistically significant.

## Results

### Pre-operative Characteristics of Patients

Under emergency conditions, 12 patients were treated with the CG technique in combination with embolization. Patients were diagnosed as ruptured type B aortic dissection in nine patients (75%), ruptured aortic arch aneurysm in two patients (17%), and traumatic transection of the aorta in one patient (8%). The percentage of endovascular procedures was 35% (12/34) for all patients with ruptured aortic arch lesions treated in our hospital. Table 1 lists the demographic data, risks for open surgery, and clinical characteristics of 12 patients receiving endovascular repair. The mean age of patients was 61 ± 12 years (range: 41–83 years). Nine patients with severe complications or previous surgical history were considered a high surgical risk and treated with endovascular procedures.

**Table 1:** Patient characteristics.

**Characteristics**	***n*/*N* (%)**
Male sex	10/12 (83)
Age, years, mean ± SD	61 ± 12
Hypertension	10/12 (83)
Smoking	3/12 (25)
**Clinical symptoms**	
Chest pain	7/12 (58)
Hemoptysis	2/12 (17)
Dyspnea	3/12 (25)
Hoarseness	3/12 (25)
Others^*^	1/12 (8)
**Risk of open surgery**	
Advanced age	3/12 (25)
Severe COPD	1/12 (8)
Ischemic stroke	3/12 (25)
Secondary infection	2/12 (17)
ITP	1/12 (8)
**Previous aortic repair**	
TEVAR	2/12 (17)
Open repair	2/12 (17)

* *Others: One case with dysphagia, cough, and wheezing*.

*COPD, chronic obstructive pulmonary disease; ITP, immunologic thrombocytopenic purpura*.

### Operative Characteristics

The treatment strategy according to entry location is presented in Table 2. The proximal ends of entry tears were <15 mm away from LSA lateral margin in eight patients (67%), were <15 mm away from LCCA lateral margin in three patients (25%), and were <15 mm away from IA lateral margin in one patient (8%). Single-chimney (SC) technique was performed in five (5/12, 42%) patients; CGs were deployed to LSA in two (2/5, 40%) patients and LCCA in three (3/5, 60%) patients. Double-chimney (DC) technique was performed in one (1/12, 8%) patient, in which CGs were deployed to LCCA and LSA. Six (6/12, 50%) patients received TC in IA, LSA, and LCCA. All CGs were parallel and along the direction of blood flow.

**Table 2:** Treatment strategy according to the location of entry.

***N***	**Location of entry**	**First operation**						**Second operation**				
		**Number of CGs**			**FL**		**Gutter**		**FL**		**Gutter**	
		**IA**	**LCCA**	**LSA**	**C**	**G**	**C**	**G**	**C**	**G**	**C**	**G**
1	Z1	1	1	1	/	/	/	/	2	/	2	1
2	Z2	1	1	1	/	/	/	/	4	/	2	/
3	Z2	1	2	1	/	/	/	/	54	10	3	2
4	Z2	/	1	/	6	/	1	/	/	/	/	/
5	Z3	/	/	1	/	/	/	/	/	2	/	2
6	Z3	1	1	1	/	/	/	/	5	1	/	1
7	Z3	/	1	/	2	/	1	/	9	/	/	/
8	Z3	1	1	1	/	/	/	/	/	8	/	2
9	Z3	/	1	1	5	1	/	1	/	/	/	/
10	Z3	/	/	2	4	4	/	1	/	/	/	/
11	Z3	2	2	1	/	6	/	2	2	/	2	/
12	Z3	/	1	/	4	2	/	1	/	/	/	/

*C, number of coils; G, volume of Onyx glue*.

### Stent Grafts and Embolic Materials Data

The intraoperative details of the two operations are presented in Tables 3, 4. The aortic stent grafts used included those by TAG (W. L. Gore & Associates, AZ, USA; *n* = 7), Valiant (Medtronic, Minneapolis, MN, USA; *n* = 4), and Zenith (Cook, Bloomington, IN, USA; *n* = 1). The mean oversizing of main stents was 116.4 ± 5.4%. In total, 12 patients were implanted with 29 CGs, included those by Viabahn (W. L. Gore & Associates, AZ, USA; *n* = 14), Sinus (Optimed, Ettlingen, Germany; *n* = 10), Fluency (C.R. Bard, Tempe, AZ, USA; *n* = 4), and Everflex (ev3 Endovascular Inc., Minn, USA; *n* = 1). CGs include covered stents and bare-metal stents (Table 3). Bare stents were used in two cases (17%), covered stents were used in nine cases (75%), and both stents were used in one case (8%).

**Table 3:** Characteristics of aortic and chimney stent grafts.

**Aortic and Chimney Stent grafts**	***N* (%)**
**Aortic stent grafts**	
TAG (W. L. Gore & Associates, AZ, USA)	7 (58)
Valiant (Medtronic, Minneapolis, MN, USA)	4 (33)
Zenith (Cook, Bloomington, IN, USA)	1 (8)
*Chimney grafts*	
**Covered stent**	
Viabahn (W. L. Gore & Associates, AZ, USA)	14 (48)
Sinus (Optimed, Ettlingen, Germany)	7 (25)
Fluency (C.R. Bard, Tempe, AZ, USA)	4 (14)
**Bare-metal stent**	
Sinus (Optimed, Ettlingen, Germany)	3 (10)
Everflex (ev3 Endovascular Inc., Minn, USA)	1 (3)

**Table 4:** The choice of access artery for embolization.

**Access artery**	***n*/*N* (%)**
**The first operation (N = 6)**	
Right femoral artery	2/6 (33)
Left femoral artery	2/6 (33)
Left brachial artery	2/6 (33)
**The second operation (N = 8)**	
Right femoral artery	4/8 (50)
Left brachial artery	3/8 (38)
Right brachial artery	1/8 (13)

A total of 108 coils were deployed in 10 patients (83%). Between 4 and 57 coils (median, 12 ± 17) were used in each patient. Five patients (50%) used coils in the first operation, in which the embolism area was filled up after coils were released one by one through a pre-road catheter. Six patients (60%) utilized coils in the second operation, during which coils were deployed through the catheter, which was selected into FL or gutters. One patient (10%) used coils in both operations. Interlock (Boston Scientific, Natick, MA, USA; *n* = 43) was most frequently used (in eight patients, 80%), followed by MWCE (Cook Medical, Bloomington, IN, USA; *n* = 65) in two patients (20%), in one of whom required 57 MWCE coils. Nine patients (75%) underwent Onyx (ev3, Irvine, California, USA) glue injection, four (44%) in the first operation and five (56%) in the second operation; the mean volume of glue injected was 5 ± 4 ml (range: 1–12). The total injection volume of Onyx was 47 ml.

### Post-operative Outcomes and Follow-Up

**Figure 3:**
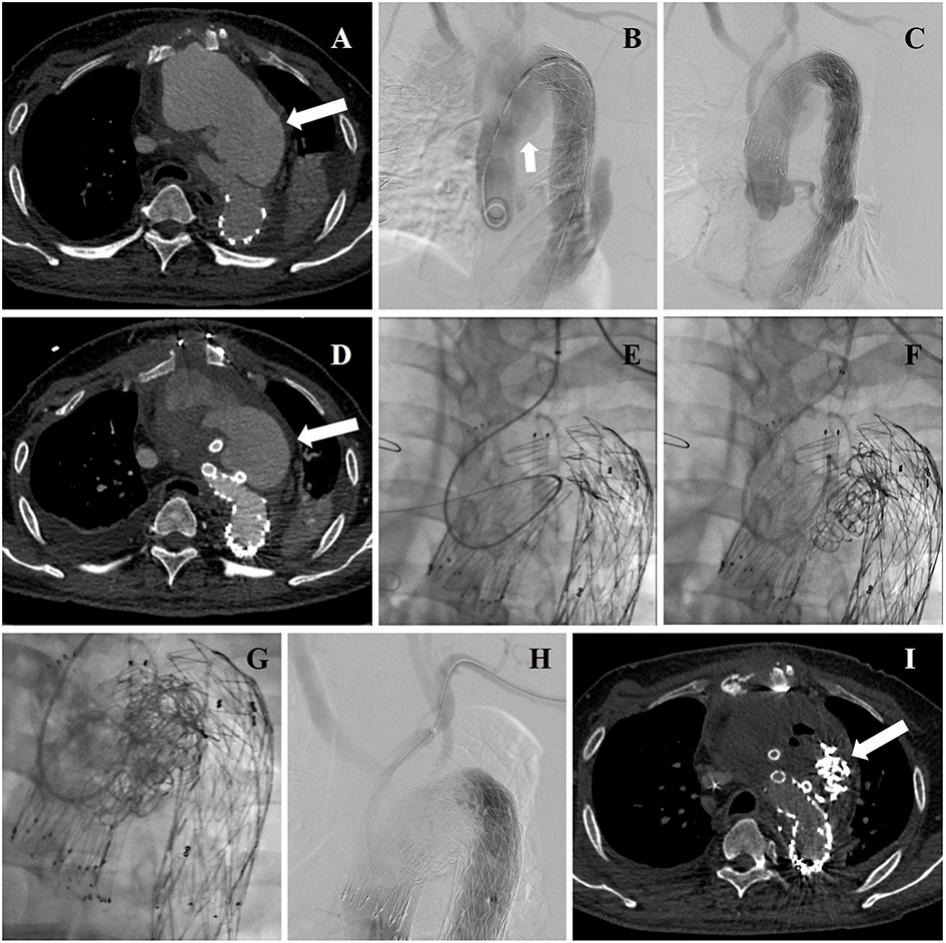
**(A)** Aortic arch pseudoaneurysm (arrow) was found after TEVAR by CTA admission. **(B)** Intraoperative DSA identified the location of lesion again (arrow). **(C)** Intraoperative aortography showing deployment of aortic stent graft and CGs. **(D)** CTA reexamination revealed post-operative EL-I (arrow). **(E)** The guidewire and catheter entered into FL through gutters by choosing left brachial artery as the access artery in the second operation. **(F)** Coils were deployed into FL and gutters. **(G)** Onyx glues were deployed into FL and gutters through a microcatheter. **(H)** A final aortography confirmed the patency of arch branches and the exclusion of EL-I. **(I)** CTA examination was performed 3 months after the second operation showing that endoleak disappeared (arrow).

All stent grafts and embolic materials were successfully deployed. Complete thrombosis of FL of the aortic arch was noted in all 12 patients. The treatment of three representative patients is showed separately in Figures 1–3. The mean interval from onset to the first operation was 2 ± 1 days (range: 1–7 days) in 12 patients. The time between the first surgery and revision was 3 ± 1 day (range: 2–7 days) in six patients. The mean endovascular procedure time of the first operation was 224 ± 87 min (range: 105–370 min), and that of the second operation was 179 ± 95 min (range: 40–360 min). All patients entered ICU after surgery for monitoring. The mean time of ICU stay was 4 ± 7 days (range: 1–25 days). All patients completed cTEVAR and embolization during one hospitalization. The average hospitalization time was 22 ± 11 days (range: 7–44 days). The mean in-hospital expense was $65,695 ± 21,625 (range: $26,419–$95,828). Two patients (17%) experienced brain lacunar infarction during hospitalization and were cured before discharge.

Eleven patients (92%) completed the follow-up. They were closely followed up for a median of 12 months (range: 1–34 months). One patient (8%) was out of contact immediately after discharge, and his status was unknown. In this cohort, no stent graft migration, component separation, or fracture occurred. During the follow-up period, no cerebral infarction, no new endoleak, and no CG stenosis or occlusion were observed. One patient with severe thrombocytopenia eventually died of a cerebral hemorrhage on the 18th day after the second operation in another hospital, leading to a 9% (1/11) 30-day mortality. Seven patients (58%) have ≥6 months of clinical follow-up time with appropriate imaging follow-up. In four (57%) of these patients, diameter stabilization was detected, whereas three (43%) experienced a significant reduction (≥5 mm).

## Discussion

Aortic arch lesions present a challenge and are at the forefront of aortic disease treatment due to their involvement of superior branch arteries (11, 14, 21–23). When aortic dissection, aortic aneurysm, trauma, or other injury factors cause rupture of aortic arch lesions, it will progress rapidly and adversely affect heart and lung function, making it one of the most critical diseases with the highest mortality rate (8, 20).

Most ruptured aortic arch lesions are associated with circulatory instability, and some lesions are secondary to open or endovascular procedures (2, 3, 16). As a result, open surgery is often difficult to perform due to its invasiveness or poor pre-operative condition of patients. Furthermore, patients who received open surgery had high mortality and a high incidence of complications (1, 20). In comparison, stent grafting is a minimally invasive surgery compared to artificial blood vessel replacement during thoracotomy (17–24). Stent grafting has been recently reported in treating ruptured aortic arch lesions. However, traditional endovascular treatment cannot reconstruct arch branches while settling aortic arch lesions. The treatment of ruptured aortic arch under emergency conditions remains ineffective (13, 20).

How to use endovascular treatment to solve this problem becomes an important subject that requires additional research. Branched stent grafts cannot be applied to rescue ruptured aortic arch disease in emergency circumstances, as they must be customized to the arch's anatomy (25, 26). Similarly, fenestrated stent grafts, pre-fenestration technique, or *in situ* fenestration technique, which are complex and require custom-made devices, have rarely been reported in recent years (5–9, 25). Although the CG technique has been extensively published in treating aortic arch lesions, it is difficult to apply in ruptured aortic arch lesions due to the risk of endoleak (10, 16, 20, 27, 28). However, the CG technique is easy to operate and does not require customizing stent grafts under emergency conditions, making it an excellent option for treating aortic arch rupture (11, 15, 24). Therefore, how to properly handle post-operative endoleak of cTEVAR becomes the focus of endovascular treatment for ruptured aortic arch lesions.

Improved coil material and design makes coil embolization a very safe procedure. Extensive literature revealed that coil embolization was employed in the endovascular treatment of many types of aortic diseases (29, 30). Most reported cases involved remedial or assisted therapeutic methods to deal with a patent FL following TEVAR or surgical intervention (29).

Onyx glue, as a permanent liquid embolic agent, has also been widely used in treating many vascular conditions (30, 31). Previous research has indicated that using coils along with Onyx glue is a safe and efficient approach to create a durable thrombogenic environment (32, 33). However, applying coil or Onyx glue to eliminate intraoperative endoleak of CG technique for endovascular treatment of ruptured aortic arch lesions has not yet been reported.

The key to embolic elimination of EL-I lies in the entry of gutter. There are three effective strategies: reserved pre-road catheter, superselection through FL, and superselection through the graft's proximal end. However, special attention should be paid during embolization to the occurrence and risk of ectopic embolism.

## Conclusions

In patients with ruptured aortic arch lesions under emergency conditions, the CG technique extends the sealing zone of thoracic aortic stent graft to a certain extent, and a thrombogenic environment in FL and gutters is created using coils and Onyx glue to settle the problem of EL-I. The creation of this thrombosis after cTEVAR may be a safe and effective treatment strategy for aortic arch rupture. In the present study, early-term patient outcomes were satisfactory; however, long-term outcomes remain to be evaluated.

## Data Availability Statement

The original contributions presented in the study are included in the article/supplementary material, further inquiries can be directed to the corresponding author/s.

## Ethics Statement

The studies involving human participants were reviewed and approved by the studies involving human participants were reviewed and approved by Informed Consent were obtained from all the patients and the study protocols were approved by the Ethical Review Board and Statistics Department of Shanghai General Hospital (No. 2016SQ271). The patients/participants provided their written informed consent to participate in this study. Written informed consent was obtained from the individual(s) for the publication of any potentially identifiable images or data included in this article.

## Author Contributions

RF and JF conceptualized and led the work. XB, YZ, and TL were contributed to the study design, obtaining funding, writing the article, and critical revision of the article. MW, ZZ, MG, and DX were contributed to the data collection, analysis, and interpretation. All authors contributed to the article and approved the submitted version.

## Funding

This work was supported by the National Natural Science Foundation of China [81770476 and 81970208], the Science and Technology Innovation Plan of Shanghai Science and Technology Commission [20S31901700], and Shanghai Rising-Star Program [20QA1408900].

## Conflict of Interest

The authors declare that the research was conducted in the absence of any commercial or financial relationships that could be construed as a potential conflict of interest.

## Publisher's Note

All claims expressed in this article are solely those of the authors and do not necessarily represent those of their affiliated organizations, or those of the publisher, the editors and the reviewers. Any product that may be evaluated in this article, or claim that may be made by its manufacturer, is not guaranteed or endorsed by the publisher.
